# A randomised controlled feasibility trial and qualitative evaluation of an early years language development intervention: study protocol of the ‘outcomes of Talking Together evaluation and results’ (oTTer) project

**DOI:** 10.1186/s40814-019-0498-2

**Published:** 2019-10-29

**Authors:** Claudine Bowyer-Crane, Dea Nielsen, Maria Bryant, Nimarta Dharni, Rebecca Heald, Chloe Storr, Josie Dickerson

**Affiliations:** 1 Department of Education, University of York, Heslington, York, YO10 5DD UK; 2 University of York, Heslington, York, YO10 5DD UK; 3 Clinical Trials Research Unit, Leeds Institute of Clinical Trials Research, University of Leeds, Leeds, LS2 9JT UK; 4 Better Start Bradford Innovation Hub, Born in Bradford, Bradford Teaching Hospitals NHS Foundation Trust, Bradford, BD9 6RJ UK; 5 BHT Early Education and Training, 16 Teasdale Street, Bradford, BD4 7QJ UK

## Abstract

**Background:**

Problems with oral language skills in childhood have been linked with poor educational, employment, and mental health outcomes. In the UK, there is increasing concern about the oral language skills of children, particularly children from areas of social disadvantage. Research emphasises the importance of the home language environment as a fundamental bedrock for the development of oral language skills. It is vital, therefore, that support is available to help families in need to provide the optimal language environment for their child. Talking Together is a 6-week home visiting programme recently commissioned by Better Start Bradford to develop parents’ knowledge of the importance of a good language environment and help to improve parent-child interactions. This study represents the initial steps in developing a definitive trial of the Talking Together programme.

**Method:**

This study is a two-arm randomised controlled feasibility study in which families referred into the Talking Together programme and consent to participate in the trial will be randomly allocated to either an intervention group or a waiting control group. We will assess the recruitment and retention rates, the representativeness of our sample, the appropriateness of our measures, and the sample size needed for a definitive trial. We will also carry out a qualitative evaluation to explore the acceptability of trial procedures for families and service providers, fidelity of delivery, time and resources for training, and barriers and facilitators to engagement with the programme. Clear progression criteria will be used to assess suitability for a definitive trial.

**Conclusion:**

This feasibility study will inform the development of a definitive trial of this home-based visiting programme, which will add to the sparse evidence base on which practitioners can draw when supporting families in need. The lessons learnt from this feasibility study will also inform the wider evaluation work of the Better Start Bradford Innovation Hub.

**Trial registration:**

The trial is registered with the ISRCTN registry: study ID ISRCTN13251954. Date of registration: 21 February 2019 (the trial was retrospectively registered).

## Background

Over the past 10 years, a number of reports have raised concerns about the oral language skills of children in the UK. In particular, it is estimated that speech, language, and communication difficulties can affect up to 50% of children from more deprived backgrounds across the UK [1]. Strong language skills underpin children’s educational achievement and have been found to be predictive of both social and scholastic success [2, 3]. Moreover, early language difficulties have been linked to poor adult mental health outcomes, particularly anxiety and social phobia [4]. There is therefore clearly a national interest in considering what can be done to support early language development, particularly in children from deprived backgrounds. Unfortunately, the recent Bercow 10 report [1] suggests that not enough is being done to address children’s language needs and the Early Intervention Foundation (EIF) recently suggested that language should be a public health issue [4]. Indeed, the EIF suggest that ‘Child language is similar to obesity and other risk factors (such as mental health and diet) in terms of its impact on children’s overall wellbeing’ [4] (page 36). Research identifies the home learning environment as the bedrock for children’s early language development [2, 5], and it is therefore vital to support families in need to foster positive parent-child interactions and supportive home environments that enrich children’s early language learning opportunities. Moreover, it is important that practitioners working with families have access to high-quality evidence-based programmes in order to ensure they can provide the most effective means of support [6]. Unfortunately, few such evidence-based programmes exist [7, 8]. While recent meta-analyses provide some preliminary evidence of the positive impact of parent-implemented training programmes [9, 10], these reviews also show that many of these studies lack robust research designs and measures of treatment fidelity, and very few of these studies are contemporary and therefore potentially out of line with current approaches to speech and language therapy [9, 10]. Moreover, none of the studies reported look at outcomes for families for whom the language of intervention is not their first language. Indeed, Tosh et al. [10] exclude studies whose population included families that have English as a second language. This paper outlines the protocol for a feasibility study of the Talking Together programme: a two-staged intervention that offers universal screening of all 2-year-old children and then provides a home-visiting programme for children at risk of language difficulties. Importantly, the programme is designed to be used in a deprived multicultural community and delivered in the families’ home language. The programme aims to develop parents’ knowledge of the importance of a good language environment and helps to improve parent-child interaction with the specific aim of supporting children’s early language development.

The Talking Together programme was developed by BHT Early Education and Training and has been running in Bradford for a number of years. The programme has recently been commissioned by Better Start Bradford, one of five ‘A Better Start’ programmes across the UK, funded by the Big Lottery, with the specific aim of improving the outcomes of children in three areas: social and emotional development, nutrition and obesity, and communication and language development through a portfolio of commissioned services. Central to the work of Better Start Bradford is the collaboration with Born in Bradford (BiB), which enables robust evaluations of the commissioned services [11] and aligns the work with health services in the city of Bradford through BiB’s position as part of the Bradford Institute for Health Research (BIHR) based at Bradford Teaching Hospitals NHS Foundation Trust (BTHFT). The feasibility study described in this protocol takes the required steps towards a full effectiveness evaluation of the Talking Together intervention by establishing the acceptability and feasibility of conducting a trial, including the use of qualitative and quantitative methods to explore implementation. This study is referred to as the oTTer project (outcomes of the Talking Together evaluation and results) and is a collaboration between researchers at the University of York, University of Leeds, and Born in Bradford, and service providers at BHT Early Education and Training, with funding from the Nuffield Foundation.

### Research aims

The aim of the oTTer project is to establish the feasibility of a definitive RCT trial of Talking Together. There are two key objectives involved in meeting this aim:

1. To assess the feasibility of conducting a trial to evaluate the effectiveness of Talking Together including the acceptability of the intervention outcome measures
2. To embed a qualitative evaluation within the oTTer trial to identify challenges with the implementation and delivery of the Talking Together programme as part of a trial

### Research questions

#### Aim 1

The research questions for aim 1 are as follows:

1. What are the recruitment and retention rates of Talking Together established by the number of participants who were identified, eligible, approached, consented, randomised, completed the programme, and followed up 6 months after baseline?
2. How representative are the trial participants compared to the wider population receiving the intervention, based on key demographic indicators?
3. What are the most appropriate outcome measures for a future definitive RCT, considering the acceptability, reliability, data quality (completeness), and responsiveness of administered measures?
4. What is the sample size needed for a definitive trial based on data on intervention completion and attrition rates, along with outcome data group differences and variability across conditions?
5. How acceptable are the intervention and trial procedures for practitioners and families, including randomisation and completion of outcome measures?

#### Aim 2

The research questions for aim 2 are as follows:

1. Was the intervention delivered with fidelity to the standardised procedures as measured by assessing the intervention content, and the frequency and duration of support received by participants?
2. What are the time and resources required to train practitioners to administer the intervention, and how do these relate to resource requirements for definitive RCT development?

## Method

The protocol has been written in line with the SPIRIT checklist [12].

### Setting/population

Better Start Bradford provides services to families in three areas of Bradford, the population of which make up approximately 12% of the entire Bradford area. These three inner-city areas are ethnically diverse and are among the most deprived both in Bradford and in England [11]. In terms of language and communication needs, recent government statistics show that 20% of children in the Bradford area do not achieve expected levels of development in communication and language compared to a national average of 18% [13]. In the Better Start Bradford areas, recent estimates suggest that these figures are higher, with approximately 23.5% of children not achieving expected levels in language and communication [14].

### Intervention

The Talking Together programme takes a two-step approach. Firstly, a universal language screening (see the “Screening measures” section below) is provided to the community by BHT Education and Training, in which the programme is successful in seeing over 65% of the eligible population. From this screening data, language development workers (LDWs; early year’s practitioners with specific training in children’s early language development) are able to identify families who may benefit from the programme based on both child factors (i.e. weak language development) and parent or home characteristics (e.g. parent-child interaction, a lack of developmentally appropriate materials in the home). This combined approach to identifying appropriate recipients of intervention is in line with the recent recommendations provided by the Education Endowment Foundation and Public Health England [6]. The intervention aspect of Talking Together draws on research emphasising the role of positive parent-child interaction in early language development and equips parents with the skills and knowledge to provide a supportive home learning environment. Talking Together currently serves a culturally diverse and deprived inner-city population, and the programme was developed to be appropriate for delivery to families from a range of cultural and linguistic backgrounds. This is a particular strength of the programme, given the growing need for interventions that can adapt to the needs of the increasingly diverse UK population.

Talking Together is delivered by trained LDWs in the child’s home on a 1:1 basis with individual parents/carers. The programme consists of six weekly, hour-long sessions followed by a review approximately 3 to 4 months after the intervention. The Talking Together programme aims to give parents/carers the knowledge, understanding, and tools to improve their child’s communication skills. Weekly sessions cover five topic areas related to improved language and communication, including what is communication, play, attention and listening, turn taking, praise and encouragement, and a final overview session. During these sessions, LDWs provide information about the week’s core topic and then engage parents in conversation about their understanding of the topic and how they address it in their home. For example, on the topic of play, the session would present factual information on the importance of play for learning, and LDWs would then discuss parents’ own experiences of play as a child and now with their own child. In addition to this, LDWs observe parents’ interactions with their child, and then use a range of techniques to highlight parents’ positive behaviours and support identification and change of less beneficial behaviours. These techniques include praising, modelling of good practice, and providing suggestions and examples for parents to try themselves. A central premise of Talking Together is that it takes a positive, child-centred approach that focuses on improving the child’s learning environment, rather than a parent-centred approach that focuses on caregiver responsibility and aptitude. This helps to ensure that conversations are constructive, and parents feel supported rather than judged in their parenting.

Practitioners are required to follow a programme manual, which contains session content for each week, activities for parents to complete between sessions, and information resources for parents. Although Talking Together is manualised and there is an expectation of fidelity to the programme, it is also personalised to be as suitable as possible to individual families. LDWs are encouraged to use their extensive training to ensure that content is engaging, relevant, and useful as possible for participants. They do this by getting to know the family’s needs and interests, and using this understanding to provide bespoke examples and activities around the core content. In addition to this, the sixth and final review session is an opportunity for the family and LDWs together to decide what content from the programme they would like to consider in more depth. The LDWs then tailor this session to be most useful for each individual family.

LDWs also take books and simple play resources/toys to sessions to give parents ideas on how to play with their child and develop communication skills. As well as delivering the session content in the home, LDWs also assess whether the home provides a good learning environment, looking for distractions that may be disruptive to learning/communication development, and advising families on how to improve the home learning environment.

#### Training and quality assurance

The service provider has robust procedures to train and support new members of staff, and monitor implementation across all staff members, to ensure fidelity. New staff members are trained over a period of several months, including participating in a range of mandatory courses and extensive shadowing of other LDWs’ practice. All staff deliver at least one full course of the intervention in conjunction with another LDW before being approved for independent practice.

With regard to quality assurance, all sessions are delivered with strict adherence to the manual and delivery guidelines, and this is monitored through regular observations of practice. All staff members receive at least two observations and evaluations of their session delivery every year by a senior LDW. Session observations are video recorded, and staff review their delivery with a senior member of staff to ensure that programme content is fully covered and practitioners’ skills are supported and developed. In addition to this, all staff participate in regular supervision with members of the senior staff, to further ensure consistency in programme delivery and staff members’ professional practice.

#### Comparator

At present, Talking Together operates a waiting list and a proportion of families therefore naturally experience a delay between referral and intervention. The maximum waiting time any family should experience under standard practice is 6 months. For the study, families allocated to the waiting control group will receive Talking Together 6 months after their baseline assessment; mirroring and not exceeding the current maximum wait time for families. At this point, they will be reassessed before they receive the Talking Together intervention as part of routine care. If at this reassessment the LDW concludes on the basis of the assessment measures that the family no longer requires Talking Together, they will be discharged from the service as is standard practice.

### Design

This will be a two-arm randomised controlled feasibility study, with an experimental group and a waiting control group. Families will be randomised following baseline data collection collected at the standard universal screening assessment delivered in the home. The families allocated to the experimental group will receive the Talking Together programme within approximately 2 weeks of allocation, while the families allocated to the waiting control group will receive the programme after a 6-month waiting period. While the average waiting time in standard practice varies depending on a number of factors, with the majority of families seen within 3 to 4 months, many families wait up to 6 months for intervention. In order that we can ensure time of data collection is comparable across the intervention and waiting control groups, we have time-locked the assessments at 2 and 6 months following pre-test thereby mirroring but not exceeding the maximum wait time. Families will be fully informed of the timeline of the project at recruitment, it will be made clear that families do not have to participate in oTTer to receive Talking Together, and full information regarding withdrawal procedures will be provided.

Feasibility and implementation outcomes will be measured using child language assessments, measures of home learning environment and parent-child relationship, and monitoring data in the form of, for example, referral rates, waiting times, attrition rates, and completion rates.

Based on the recent MRC guidance [15], our qualitative evaluation is underpinned by the conceptual framework for implementation fidelity by Hasson [16], e.g. focusing on measures of adherence and potential moderators. This framework is embedded within our wider implementation evaluation work [17]. Qualitative data will be collected through interviews with service providers to explore any issues with running the programme as part of a trial. These interviews will take place immediately after follow-up assessment has been completed for the intervention group. In addition, we will invite a sample of oTTer families to take part in structured interviews to explore their experiences of participation in the oTTer project. Topic guides will be based on the Theoretical Domains Framework, a widely used framework in behaviour change and implementation research [18, 19]. While the conceptual framework for implementation fidelity is the overall evaluation framework and outlines the key research questions, use of the TDF allows an in-depth exploration of the barriers and facilitators of implementing the trial.

While this research is being carried out within a diverse community, interpreters will not be used in the delivery of the Talking Together programme or any of the data collection. The eligibility criteria state that families must speak one of either English, Urdu, or Punjabi to their child and we have LDWs, RAs, and PDRAs who can speak to the families in their home language without the use of interpreters.

### Eligibility

#### Families

##### Inclusion

The inclusion criteria for families are as follows:

- Families must live in the Better Start Bradford reach area.
- Families must have a child aged two to two and a half years when referred into the Talking Together programme following a screening assessment by a LDW.
- Families must be willing to receive the intervention delivered by a LDW in their home.
- Families need to allow the intervention programme to be delivered with the same primary parent/carer at each session. More than one parent/carer may be present at the sessions, but there must be one consistent parent/carer who is present at all sessions.
- Families must be willing to be randomly allocated to treatment or control group and consent to additional data collection if allocated to the control group.
- Families must speak English, Urdu, or Punjabi as the primary language with their child established via assessment and observation by a language development worker.

##### Exclusion

The exclusion criteria for families are as follows:

- Children who have any known significant developmental disorder or sensory impairment.
- Families who are referred into the Talking Together programme at the request of external bodies, i.e. safeguarding authorities.
- Families where the primary carer/parent to whom the intervention programme will be delivered may vary from session to session.

#### Service providers

##### Inclusion

The inclusion criterion for service providers is as follows:

- Staff must be LDWs or senior staff involved in the administration and delivery of the Talking Together programme at BHT Early Education and Training.

##### Exclusion

The exclusion criterion for service providers is as follows:

- Staff who were not involved with Talking Together in the Better Start Bradford reach area during the time of recruitment to the trial.

### Recruitment and randomisation

#### Sample size

**Fig. 1:**
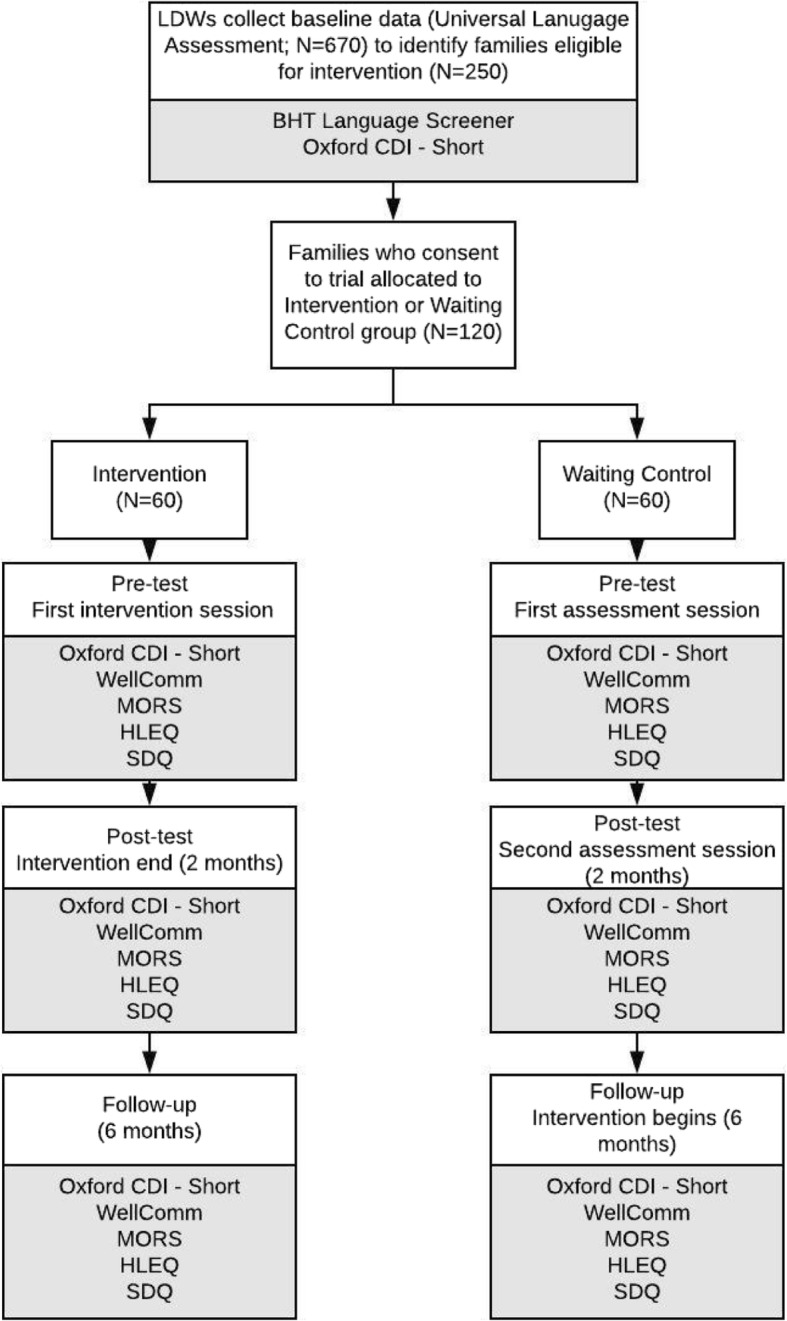
Flow chart of feasibility study of Talking Together programme

**Fig. 2:**
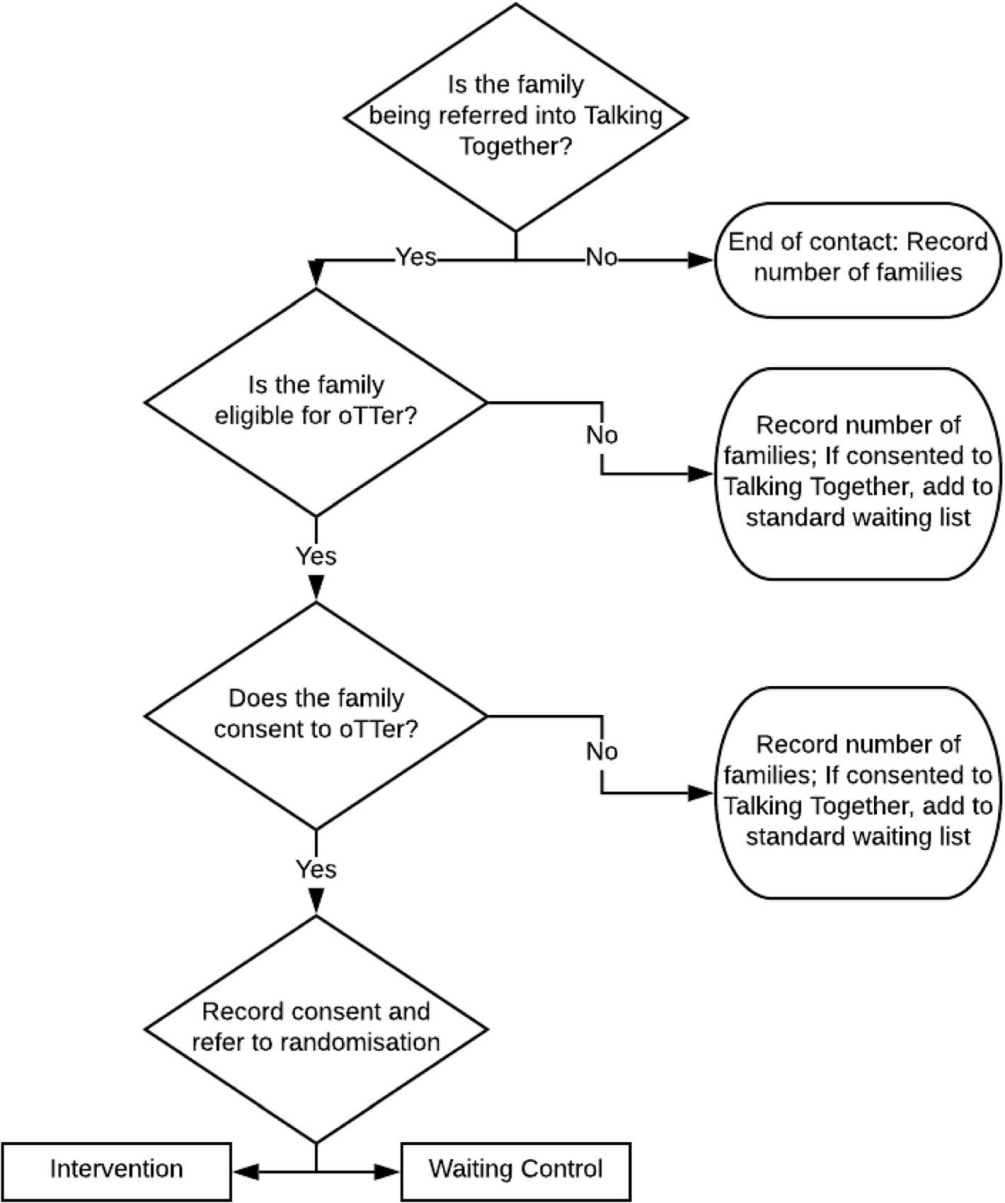
Procedure for recruitment and consent in the oTTer feasibility study

Based on existing recruitment data, over the 9-month period of recruitment, we would expect BHT Education and Training to screen approximately 670 families and refer approximately 250 families to the Talking Together programme, although not all of these families will be eligible for the trial or consent to take part. Recommendations for feasibility studies suggest that at least 30 participants per group will allow sufficient precision when estimating study summary measures [20, 21]. As such, our minimum required sample size is 60 families. However, we have set a desired sample size of 120 families, which is in excess of the recommended 60 participants for a feasibility study [14, 15], to allow for attrition and provide more confidence that a future definitive trial can be successfully conducted (see Fig. 1) [14]. Currently, all families within Better Start Bradford with children of 2 years of age are offered a language screening home visit. For the purposes of recruitment, the LDWs will provide families with an information sheet and consent form to take part in the trial at the end of these language screening visits if the family is going to be referred to Talking Together and meets the oTTer eligibility criteria. Families who consent to take part in the trial will then be randomly allocated to the intervention group or the waiting control group (see Fig. 2).

Randomisation (1:1) will be carried out by a member of the data management (DM) team within BiB. Statistical software package STATA (‘ralloc’ command) will be used to generate a random list stratified by (a) whether the family was within a specific Children’s Centre reach area, (b) language of delivery (English or not), and (c) whether two or more children will be present during delivery of the intervention, using a block size of eight. Referrals from the LDWs with informed consent to take part in the trial will be entered onto SystmOne, a secure healthcare system managed by BTHFT. Once a week, the DM team will carry out randomisation and enter the allocation details onto SystmOne. The deputy language development programmes manager will then access the randomisation details and allocate families to the research assistants and LDWs as appropriate so that a schedule of assessment and intervention can be devised. It is not possible to fully blind the LDWs, RAs, and post-doctoral research assistant (PDRA). The LDWs and RAs will carry out the assessments with the intervention and control group respectively and therefore cannot be blinded to group membership. The PDRA will only have access to anonymised data, but will know the group allocation of all anonymous participants for the purposes of monitoring trial procedures and data analyses.

#### Qualitative evaluation

For interviews, we will invite all LDWs and a purposive sample of oTTer families to participate. For service provider interviews, we are aiming to recruit a minimum of 50% of LDWs along with all members of the senior staff team involved with the Talking Together programme. For family interviews, we are aiming to recruit 20 to 30 oTTer families (10–15 from the intervention arm, and 10–15 in the waiting control arm) who are representative of the range of diversity in the sample. A purposive sampling framework based on key characteristics of families (e.g. main language spoken to child, ethnicity, group allocation, number of children in home) will be used to identify and invite families in the oTTer study to take part in interviews. Invitations will be made via the LDWs during a routine visit. Consent will be taken at the point of invitation, and it will be made clear that families will be contacted by a RA to arrange an interview. During this follow-up contact, families will be given the opportunity to withdraw consent to take part in the interview, and they will again be reminded of the right to withdraw prior to the start of the interview itself. Interviews will take place in the families own home.

All LDWs and senior staff with responsibility for Talking Together will be approached to take part in interviews once recruitment has finished. All LDWs (approx 15) and senior staff (*n* = 3) will receive a letter from the research team inviting them to participate in interviews. Interviews will be scheduled in discussion with the deputy language development programmes manager to ensure maximum participation with minimum disruption to service. The interviews will be carried out by a PDRA who has not previously worked with the BHT team and who can complete the interviews without the support of an interpreter.

### Measures

#### Primary outcomes

Our primary feasibility outcome is recruitment to the trial. We will record the following:

- Number of parents receiving the universal screening
- Number of parents eligible to receive Talking Together
- Number of parents offered Talking Together
- Number of parents accepting Talking Together
- Number of parents eligible for the feasibility study
- Number of parents not eligible for the feasibility study and reason why
- Number of parents approached to take part in the feasibility study
- Number of parents consenting to take part in the feasibility study

#### Secondary outcomes

In order to fully explore feasibility, there are a number of secondary outcomes:

1. Training and QA
  - Length of time taken to train staff to deliver Talking Together
  - Number of staff trained to deliver Talking Together
  - Programme delivery quality assurance (collected routinely by BHT)
2. Data collection
  - Number, proportion, and timing of parent withdrawals from the Talking Together intervention and feasibility study and reasons for withdrawal
  - Number and proportion of parents with quantitative data at each time point
  - Missing item level data on intervention outcome measures at each time point
3. Sample size estimation
  - Difference between arms and 95% confidence intervals for the two main child language measures (see the “Screening measures” section and “Intervention outcome measures” section below) at 6-month follow up
4. Adherence and moderators
  - Based on Hasson [15], our evaluation will use quantitative and qualitative data to look at adherence and potential moderators. To measure adherence, we will collect data on frequency and number of sessions delivered, and completion rate of families. To explore potential moderators, we will obtain feedback on acceptability of trial procedures provided by parents, LDWs, and senior staff at BHT via interviews; feedback on the acceptability of the programme provided by parents via interview; feedback on ease of delivery of the programme provided by service providers via interview; and data on implementation fidelity through evaluation of quality assurance records.

#### Screening measures

The Universal Language Assessment (baseline) is comprised of two measures, which are used in conjunction with observation of the home and parent-child interaction to inform LDWs’ judgement about referral to the intervention:

1. BHT Language Screener Measure: this assessment was created by BHT in conjunction with speech and language therapists as well as academic partners. It is comprised of ten statements about children’s current language skills scored on a 3-point scale (the child does not do this yet, does this sometimes, does this often). LDWs also record their specific reason for referring into the programme (language and communication, child behaviour, parent behaviour, home learning environment, supporting a family with complex needs).
2. The Oxford Communication Development Inventory-Short (CDI-Short) [22]: a validated measure of early language development comprised of 100 vocabulary items. Parents complete this with the support of the LDW and indicate words the child can (a) say and (b) understand. This measure is used as an indication of conceptual vocabulary, and parents are asked to indicate which words their child can say and understand in any of the languages they speak. It is a parent-report measure that takes approximately 10 min to complete

#### Intervention outcome measures

One of the goals of this feasibility study is to establish the most suitable measures for use as intervention outcomes. After an extensive search we selected two measures that were suitable in terms of ease of use, time taken to administer, and suitability for use with multilingual families. We recognise the limitations inherent in direct translations of language measures but have selected measures that we are confident have attempted to do this as reliably as possible. All outcome measures will be administered at pre-test, post-test, and follow-up.

Two outcomes will be evaluated to determine which is most suitable as a primary outcome for future definitive trial at 6 months follow-up:

1. The Oxford Communication Development Inventory-Short (CDI-Short) [22]: This assessment is re-administered as an outcome measure.
2. The WellComm language assessment [23]: a validated, direct, and objective assessment of children’s language development. The measure is completed through questions with parents and observation of the child by the LDW. The assessment can be directly translated into Asian languages up to section 5 (https://www.gl-assessment.co.uk/support/support-videos/wellcomm-support-videos/). Where families are receiving the intervention in a language other than English, the LDW will deliver the WellComm assessment in the primary language of the child.

Three additional measures will be evaluated for feasibility as potential secondary outcomes:

1. Maternal Object Relations Scale (MORS) [24]: this is a validated self-report measure which assesses parent/carer and child relationships and attachment. LDWs help parents complete the measure, and it will be administered in the dominant language of the parent.
2. Home learning environment questionnaire (HLEQ) [25]: an indicator of the types and frequency of activities in the home shown to be predictive of children’s later language skills. LDWs help parents complete the measure, and it will be administered in the dominant language of the parent. This measure has not currently been validated.
3. Strengths and Difficulties Questionnaire (SDQ) [26]: this is a validated measure of children’s emotional and behavioural adjustment. The scale consists of five subscales: emotional symptoms, conduct problems, hyperactivity/inattention, peer relationship problems, and prosocial behaviour. For the purposes of this study, only the hyperactivity and conduct subscales are administered. It should be noted that while validated as a whole measure, the SDQ not been validated to be used in this way. However, a pragmatic decision was made to reduce the measure to these two subscales as the entire SDQ is too long to administer alongside the other quantitative measures. LDWs help parents complete the measure, and it will be administered in the dominant language of the parent.

### Data collection

**Fig. 3:**
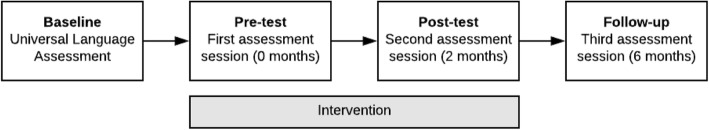
Assessment points and timings for outcome data collection and how they correspond to the timing of the intervention

As our outcome measures are administered routinely by LDWs, and in order to avoid repeat assessments, data will be collected by both LDWs and research staff. Baseline data will be collected during universal language assessment sessions by LDWs as standard practice. Pre-test and post-test data will be collected by LDWs for the experimental group and RAs for the waiting control group. Six-month follow-up assessments in both treatment arms will be carried out by trained RAs accompanied by a LDW familiar with the family to maintain consistency and make clinical judgements about the need for ongoing input. LDWs and RAs will receive the same training in administering the assessments and will carry out reciprocal observations of assessments. The timeline for assessment can be seen in Fig. 3.

Qualitative interviews with the service provider and families will be collected by a PDRA not responsible for collecting any other trial data. We will carry out interviews with service providers after recruitment to the trial has ended. All LDWs and members of the Talking Together management team will be invited to take part in these interviews. We will invite a purposive sample of oTTer families to participate in interviews at the end of their follow-up period (approximately 6 months after the baseline assessment). Interviews will be carried out in person or by phone at the convenience of the families. All qualitative work will be completed 24 months after recruitment commenced.

### Statistical analysis

No interim analysis is planned. Statistical analyses will be carried out after all outcome data has been collected. Feasibility objectives and acceptability of intervention outcome measures will be assessed using descriptive analysis, focusing on confidence interval estimation, rather than formal hypothesis testing. To assess the suitability of the intervention outcome measures for use with multilingual families, we will compare quality and responsiveness of the measures in families who do and do not speak English as their primary language.

### Qualitative analysis

Qualitative data from interviews will be transcribed and checked for accuracy against the original audio recordings. Anonymised transcripts will be analysed using the Nvivo data management programme (NVivo qualitative data analysis software; QSR International Pty Ltd) and analysed following a two-stage approach. In stage 1, transcripts will be coded to the domains of the TDF to extract the barriers and facilitators of implementing the trial from the perspective of service providers and families, followed by an inductive analysis of the themes within each domain. We will also explore any patterning of themes by individuals’ ethnicity, socioeconomic circumstances, and English language ability. Transcripts will be coded systematically and iteratively until saturation is achieved. Ten percent of transcripts will be coded by a second researcher to ensure reliability of the coding framework.

### Progression criteria

Progression to definitive trial will be informed by the results of three main progression criteria, based on Avery et al. [27]. A traffic light system will be used with red as an indication not to progress to trial and green indicating progression to trial. Amber indicates potential progress to trial with careful consideration of why these criteria did not reach green.

1. Recruitment—we anticipate a minimum of 60% of families identified and offered the intervention will be eligible to participate in the trial, and a minimum of 50% of these eligible families will consent to participate in the trial. These numbers will be assessed cumulatively over the course of the recruitment phase. Progression criteria are as follows:
  - Eligibility—60% and above = green, 50–60% = amber, and below 50% = red
  - Consent—50% and above = green, 40–50% = amber, and below 40% = red
2. Protocol adherence—families will be administered the quantitative assessment measures at specific time points, ensuring that assessments of the waiting control group align with those of the intervention group. Feasibility of running a trial using this design is dependent on the ability to adhere to the following timeline: pre-test within 1 month of Universal Language Screener, post-test 6 to 10 weeks following pre-test, and follow up at 5.5 months to 6.5 months after pre-test. Progression decisions will therefore be based on percentage adherence rates, i.e. 80% = green, 60–80% = amber, and less than 60% = red.
3. Attrition rates—based on previous reported attrition rates for Talking Together, we have calculated a predicted attrition rate over the course of the trial. Progression decisions will be based on the proportion of the recruited sample that attends the 6-month follow-up, i.e. 80% = green, 70% = amber, and below 70% = red.

### Data monitoring and confidentiality

#### Quantitative outcome data and monitoring data

BHT Early Education and Training, BiB, and Better Start Bradford have a data sharing agreement. Where participants have consented to data sharing, data will be transferred to BiB automatically through the SystmOne database, i.e. SDQ, MORS, home learning environment, CDI, and WellComm, as well as recruitment and monitoring data. A data sharing agreement is also in place between BiB and the University of York, allowing BiB as data controller to share anonymised data with the PI and research team as data processors. Data quality will be monitored as a standing item on the monthly oTTer meeting agenda.

#### Qualitative data

RAs will upload audio recordings of interviews to password protected NHS laptops. Once uploaded, the recordings will be held in BiB on a secure computer server and deleted from the laptop and audio recording equipment. Transcripts will be anonymised before analysis and stored on a central secure computer at BiB. Only anonymised scripts will be shared with the research team for analysis and recordings will be destroyed after transcription.

### Project oversight

The programme management team is made up of the principal investigator, co-investigators, PDRA, data manager, and research assistants. The team will meet monthly to monitor the progress of the project, discuss challenges, set priorities, and talk about next steps.

The proposed project sits within the existing work of Better Start Bradford and BiB, and will therefore come under the purview of an established structure of expert and stakeholder advisory groups [11]. As such (and given the low risk nature of the research), a separate Data Monitoring and Ethics Committee is not required. This network of existing groups will enable the research team to ensure that the project is embedded in the wider context of Better Start Bradford and BiB.

In addition, a bespoke advisory group will be convened with oversight for this specific project. This group will be made up of members from the existing advisory groups related to the wider work of BiB and Better Start Bradford’s language and communication work, and parent representatives from the community. This group will meet with the research team biannually over the course of the project and advise on key methodological decisions, outputs, and dissemination. Meetings will be held in Bradford to ensure community members are able to attend. We will also seek advice from relevant members of the advisory board outside of these meetings as appropriate.

### Publication policy

The study falls under the rubric of the Better Start Bradford Innovation Hub and will therefore use the Better Start Bradford Innovation Hub publication policy that follows the BMJ rules on authorship and contributorship to guide decisions around publications (https://www.bmj.com/about-bmj/resources-authors/article-submission/authorship-contributorship). The publication policy is held at BiB and has been agreed by all BiB collaborators working on the Better Start Bradford programmes.

### Trial status

The trial is open and has been recruiting since October 2018. The trial was registered with ISRCTN in February 2019.

## Discussion

Recent reports have raised awareness of the need for increased early support for children with speech, language, and communication needs [1, 4]. Oral language difficulties have been linked with poor educational, employment, and mental health outcomes [4]. Research suggests that a strong foundation in language is fundamental to later language skills, and a corollary of this is that a communication-rich home language environment is key. However, the evidence for programmes that support parents in providing this strong foundation is lacking [6]. This programme of work is the first step towards developing a definitive randomised controlled trial to evaluate the effectiveness of one such programme: Talking Together. Designed and delivered by BHT Early Education and Training, the programme is one of a suite of services provided as part of the Better Start Bradford project and aims to improve children’s language outcomes through improving parent-child interactions. This study aims to inform the feasibility of delivery of a future definitive trial and as such will not provide evidence of effectiveness. However, it will provide invaluable information regarding the feasibility of using random allocation without adversely impacting on service delivery. It will identify the most effective measures to be used as primary and secondary outcomes (and how they can be administered), and through the qualitative work with service providers, it will identify any issues of implementation both for the service as a whole and for the service as part of the trial. Our qualitative work with families will help to identify barriers and facilitators to engaging with the Talking Together programme from an ethnically and socioeconomically diverse community. This increased understanding will lead to more informed commissioning decisions and service provision within Bradford and elsewhere, as well as providing the foundation for a large-scale RCT of the Talking Together programme.

## Data Availability

Study materials are available through the oTTer website: https://otterproject.wixsite.com/otter. Data from the feasibility trial will be available after final evaluation and publications of the study and can be requested following procedures described on the Born in Bradford website: www.borninbradford.nhs.uk.
